# Impacts of information and communication technologies on nursing care: an overview of systematic reviews (protocol)

**DOI:** 10.1186/s13643-015-0062-y

**Published:** 2015-05-23

**Authors:** Geneviève Rouleau, Marie-Pierre Gagnon, José Côté

**Affiliations:** Faculty of Nursing, Laval University, Quebec, Canada; Research Center of the Centre Hospitalier de l’Université de Montréal, 850 rue St-Denis, Tour St-Antoine, third floor, Door S03-422, Montreal, QC H2X 0A9 Canada; Research Center of the Centre Hospitalier Universitaire du Québec, St.-François d’Assise Hospital, 10 rue de l’Espinay, D6-734, Quebec City, QC G1L 3L5 Canada; Faculty of Nursing, Université de Montréal, Door 5099, Pavillon Marguerite-d’Youville, C.P. 6128 succ. Centre-ville, Montréal, Québec H3C 3J7 Canada

**Keywords:** Information and communication technology, e-Health, Telehealth, Nursing practice, Nursing care, Nursing sensitive outcomes, Overview

## Abstract

**Background:**

Information and communication technologies (ICTs) used in the health sector have well-known advantages. They can promote patient-centered healthcare, improve quality of care, and educate health professionals and patients. However, implementation of ICTs remains difficult and involves changes at different levels: patients, healthcare providers, and healthcare organizations. Nurses constitute the largest health provider group of the healthcare workforce. The use of ICTs by nurses can have impacts in their practice. The main objective of this review of systematic reviews is to systematically summarize the best evidence regarding the effects of ICTs on nursing care.

**Methods/design:**

We will include all types of reviews that aim to evaluate the influence of ICTs used by nurses on nursing care. We will consider four types of ICTs used by nurses as a way to provide healthcare: management systems, communication systems, information systems, and computerized decision support systems. We will exclude nursing management systems, educational systems, and telephone systems. The following types of comparisons will be carried out: ICT in comparison with usual care/practice, ICT compared to any other ICT, and ICT versus other types of interventions. The primary outcomes will include nurses’ practice environment, nursing processes/scope of nursing practice, nurses’ professional satisfaction as well as nursing sensitive outcomes, such as patient safety, comfort, and quality of life related to care, empowerment, functional status, satisfaction, and patient experience. Secondary outcomes will include satisfaction with ICT from the nurses and patients’ perspective. Reviews published in English, French, or Spanish from 1 January 1995 will be considered. Two reviewers will independently screen the title and abstract of the papers in order to assess their eligibility and extract the following information: characteristics of the population and setting, type of interventions (e.g., type of ICTs and service provided), comparisons, outcomes, and review limitations. Any disagreements will be resolved by discussion and consensus involving the two reviewers or will involve a third review author, if needed.

**Discussion:**

This overview is an interesting starting point from which to compare and contrast findings of separate reviews regarding the positive and negative effects of ICTs on nursing care.

**Systematic review registration:**

PROSPERO CRD42014014762.

## Background

Information and communication technologies (ICTs) embody all digital technologies that support the electronic capture, storage, processing, and exchange of information in order to promote health, prevent illness, treat disease, manage chronic illness, and so on [1, 2]. In the health sector, ICTs refer to a set of projects or services that allow for remote care (telehealth), interdisciplinary clinical support, as well as knowledge transfer [3]. The use of ICTs has the potential to promote patient-centered healthcare at a lower cost, improve quality of care and information sharing, educate health professionals and patients, encourage a new form of relationship between patients and their health providers, reduce travel time, etc. [1, 4, 5]. Despite these well-known advantages, the implementation of ICTs in practice remains difficult and involves changes at different levels, including with respect to patients, healthcare providers, and healthcare organizations [6]. Nurses constitute the largest health provider group of the healthcare workforce [7, 8] and as such represent an important target for the ICT implementation process. Nurses are compelled to deal with the introduction of ICTs within nursing care, such as telecare technology, which can have impacts on nursing practices [9]. These technologies change the notions of place and presence and create distance between nurses and patients. Consequently, direct care cannot be provided in the traditional way (face-to-face) that nurses are used to [10].

Although there are several systematic reviews on the effects of ICTs on healthcare professional practices [11–13], most of them focus on physicians [14, 15]. Our preliminary searches found only three reviews that specifically targeted nurses. One Cochrane review assessed the impact of different nursing record systems on nursing practice and healthcare outcomes [16]. Urquhart et al. [16] defined nursing record systems as the record of care that is planned and provided to patients by nursing staff or by other healthcare providers (including nursing students) under the guidance of qualified nurses. In their review of nine studies, four [17–20] involved computerized nursing records systems. These systems were compared to manual (paper) nursing care planning. Spranzo [19] did not find significant results on nursing practices (i.e., nurse sickness, overtime, or job turnover). Daly and collaborators [18] pointed out that in the computerized group, a little more nursing diagnosis had been carried out. Some differences were found in the number of recorded nursing interventions (*p* = 001) and activities (*p* = 0.007). Ammenwerth et al. [17] reported that the time spent planning and documenting tasks was longer with the computerized system. Bosman et al. [20] found that the use of an intensive care information system (ICIS) among patients after cardiothoracic surgery impacts nursing activities. The duration of the admission procedure was longer in that group (regarding the registration of patient data), but in the period following admission (registration phase), the use of ICIS decreased the time nurses spent for documentation by 30 %. This time was reallocated for patient care. However, the effects of changes regarding nursing record systems on nursing practice and on patient outcomes were modest, and most of the studies targeted documentation time to perform nursing tasks, which is a small part of the nursing practice.

The other two systematic reviews concerned the use of electronic health records by nurses. The mixed review method by Stevenson et al. [21] included five studies (two quantitative and three qualitative studies) and highlighted nurses’ experience of using electronic patient records (EPR) in their practice. Nurses expressed their dissatisfaction regarding EPR systems for many reasons: they did not support their everyday clinical practice because they lacked patient overview, they did not support individualized care, they were not user friendly, and they were not always bedside accessible. Also the computer systems were considered unreliable. Another systematic review [22] examined the impact of electronic health records (EHR) on time efficiency of physicians and nurses from a total of 23 studies (11 of these studies assessed time efficiency among nurses). The main findings pointed out that using bedside terminals and central station desktops saved 24.5 and 23.5 % of the time nurses spent documenting during their shift.

These three reviews were published between 2005 and 2010, targeted specific technologies and showed modest impacts on various aspects of nurses’ practice. In general, these practices were not well defined in the reviews, and there was no conceptual framework enabling reflection on the way ICTs could influence specific aspects of nursing practices and nursing care. The knowledge generated from these reviews remains sparse because each technology is used in a specific utilization context and for a particular task (e.g., documentation, data collection, nursing diagnosis); therefore, each ICT application may impact distinct dimensions of nursing care.

### Objectives

Considering the lack of an integrated body of knowledge on the effects of ICTs on nursing care, we plan to conduct an overview of systematic reviews to reach the following objectives:

- Systematically summarize the best evidence that comes from systematic reviews regarding the effects of ICTs on nursing care.
- Explore whether specific ICTs (such as electronic health records, telemonitoring, telecare), their characteristics (e.g., mode of delivery, dosage, frequency of use), and their usage purpose (e.g., monitoring, assessment) may have an impact on nursing care.

### Why it is important to do this overview

Overviews of systematic reviews are a good way to derive the best available evidence in a single document to afford broad, cumulative statements that summarize the existing evidence on the effectiveness of interventions [23]. A comparative analysis of different ICT applications would provide a first attempt to map the potential impact of these applications on nursing care.

According to Moher et al. [24], systematic reviews can vary according to their quality, complexity and length, and are published in a variety of journals. Among the reviews retrieved, one is a Cochrane review [16] and others are non-Cochrane reviews [21, 22]: these two kinds of reviews might be different, considering the methodological rigour underlying Cochrane reviews and their regular updates [25]. Thus, it is essential to take into account the methodological quality of the reviews when interpreting their results.

## Methods/design

### Criteria for considering reviews for inclusion

The scope for the overview has been formulated with the participants, interventions, comparisons, outcomes (PICO), which is helpful and form the basis to establish eligibility criteria, with this additional component, the types of study design [26].

#### Types of reviews

We will include all types of reviews that exhibit these key characteristics:

- evaluate the influence of ICTs used by nurses on nursing care, which include nurses’ practice environment, nursing processes, nurses’ professional satisfaction, and nursing sensitive outcomes (patient outcomes);
- were published in English, French, or Spanish between 1995 and 2015;
- state a methodology (a “Methods” section) with explicit eligibility criteria;
- have systematic research strategies to identify selected reviews; and
- provide a systematic presentation and summaries of the characteristics and findings of the included reviews [25].

#### Types of participants

- Registered nurses (according to the professional legislation of each country). These nurses work in different settings, such as hospitals, outpatient clinics (ambulatory care), the community, long-term care centers, etc. We will include reviews that target nurses and other groups (ex: physicians), as long as it is possible to differentiate nurses and to extract these relevant participants’ data.
- Nurses in training or nursing students will be included only if they provide direct care to patients.
- Patients receiving care from qualified registered nurses through the medium of at least one type of ICT.

#### Types of interventions

We will consider all types of ICTs, which are digital devices/media used by nurses as a way for providing healthcare. Mair et al. [27] suggested dividing e-health (i.e., “the use of emerging information and communications technology, especially the Internet, to improve or enable health and healthcare [28]”) into four domains: management systems, communication systems, computerized decision support systems, and information systems. Each of them will be explained.

1. Management systems. They are computer-based systems for acquiring, storing, transmitting, and displaying patient administrative or health information from different sources that can support administrative or clinical activities. Management systems include ICTs such as electronic health records and personal (patients) health records.
2. Communication systems. They are telecommunication systems used when users are distant in space and/or time. This kind of communication takes place in a synchronous or asynchronous way, between health professionals or between health professionals and patients or carers. It involves a targeted sharing of information between specific individuals or individuals who play distinct roles for diagnostic, management, counseling, educational, or support purposes. There are a wide range of communication systems, from e-mail and smart phones through telemedicine and telecare systems.
3. Computerized decision support systems. These systems refer to a computer-based system, which is automated and aims to support health professionals in practicing within clinical guidelines and care pathways or providing best evidence-based care. These kinds of systems are usually operated in real time and involve decision support that comes from artificial intelligence (for example, a software program) rather than a person.
4. Information systems. These systems are defined by the use of internet technology to attain access to different information resources, such as health and lifestyle information. The information remains at a general level, and it is not tailored to specific individual needs. Web-based resources and e-health portals for retrieving information are applications of information systems.

Mair et al. [27] pointed out that e-health services can be categorized as belonging to more than one domain. However, this categorization is clear and understandable, so it can be used to map the type of intervention that will be included in the overview.

We will exclude the following types of ICTs:

- nurse management systems, which are purely administrative and designed for the management of human resources and working conditions (e.g., scheduling) and nursing staff maintenance (such as retention);
- educational systems, for example, e-learning initiatives used for the training of nursing students, unless they are applied to direct patient care; and
- telephone systems, because according to most definitions of ICTs [1, 2], they are not digital technologies and cannot support the electronic capture, storage, processing, and exchange of information.

#### Types of comparisons

The following comparisons will be made: (1) ICT in comparison with usual care/practice (i.e., any interventions that are already been provided in the health care system), (2) ICT compared to any other ICT, (3) ICT versus other types of interventions (for example, face-to-face intervention).

#### Types of outcome measures conceptualized as nursing care

**Fig. 1:**
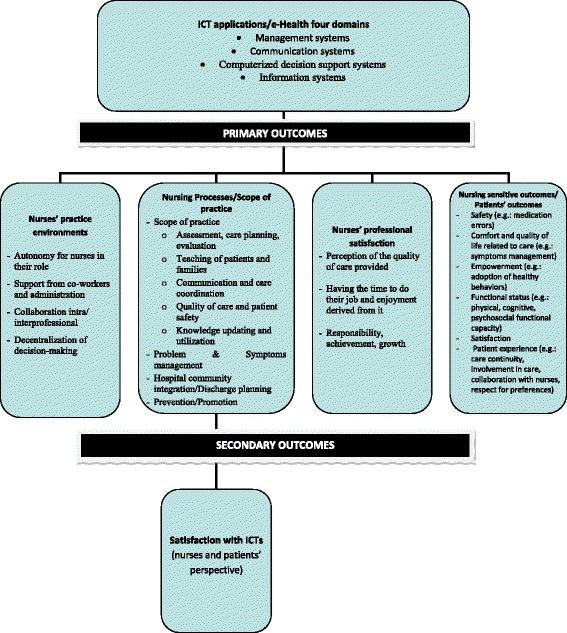
Conceptualization of the primary and secondary outcomes of interest. The conceptual framework, adapted from Dubois et al. [29] and d’Amour et al. [30], is used to conceptualize some of the dimensions of nursing care that have the potential to be influenced by ICTs

The conceptual framework that we use to illustrate how ICT interventions influence nursing care and can impact health outcomes is based on an organizational model, the Nursing Care Performance Framework [29], from which we integrated dimensions of nursing activities [30], which position the full scope of nursing practices. This model [29] illustrates how the interplay between resources or the overall structure (nursing staff) and the processes (e.g., nursing processes, practice environment, nurses-patient interaction) can produce changes in patients’ conditions. These changes can be measured through “nursing sensitive outcomes” (i.e., patient outcomes, such as patient safety, quality of life). Figure 1 shows the conceptualization of outcomes of interest. Based on the conceptual framework (Dubois et al. [29]), ICTs must have an impact on nursing practice to reverberate on patients’ outcomes. If the first condition is not fulfilled, then the impacts on patients’ outcomes do not depend on the usage of ICTs by nurses and are excluded from the review. It means that we will include patients’ outcomes as primary outcomes as long as they fall within the usage of ICTs by nurses.

#### Primary outcomes

Primary outcomes include nurse practice environment, nursing processes/scope of nursing practices, nurses’ professional satisfaction, and patients’ outcomes (nursing sensitive outcomes). The ability to perform nursing interventions/activities (nursing processes and scope of practice) is linked with organizational processes that define the nurse practice environment and can have a mediation effect on outcomes [29, 31, 32]. We believe that ICTs can influence nurses’ working environment and have the potential to impact on professional satisfaction, for example, the quality of care as perceived by nurses. Nursing sensitive outcomes (or patients outcomes), such as patient safety, comfort, and quality of life related to care, empowerment, functional status, satisfaction, and patient experience will be included as primary outcomes if they depend on the usage of ICTs by nurses. Nursing sensitive outcomes [7, 8] are “outcomes affected, provided, and or/influenced by nursing personnel” ([29] p.14). We included the indicator “patient experience” as being part of the nursing sensitive outcomes, even if the authors did not conceptualize it that way. We decided to regroup all patient outcomes under the same “outcome category.” According to Dubois et al. [29], “patient experience can be perceived as the result of the clinical (and organizational) processes that should optimally ensure that patients were provided the right nursing care, at the right time, and in the right way for them” (p.14). This is a way to assess the acceptability and appropriateness of nursing care, from the patient’s point of view. Care continuity (including care coordination, collaboration), involvement of patients and their families in nursing care (self-care/information, education), and responsiveness (respect for their preferences, quality of communication, and degree of comprehensiveness of care) are components of the patient’s experience.

The conceptualization of outcomes (i.e., nursing care), as illustrated in Fig. 1, will be redesigned (mapped in a different way) according to the results extracted from selected systematic reviews. For example, it is possible that professional satisfaction will be defined with different indicators in the systematic reviews from those presented in Fig. 1.

If these primary outcomes are met then the secondary outcomes will be included.

#### Secondary outcomes

Secondary outcomes will include satisfaction with ICT, according to the patients and nurses’ perspective.

### Search methods for the identification of reviews

We will search publications through relevant electronic databases, such as MEDLINE, CINAHL, EMBASE, PUBMED, and Web of Science (*Web of knowledge*). We will also search for the following sources, which contain systematic reviews and overviews of publications: *Cochrane Database of Systematic Review*, *Database of Abstracts and Reviews* (*DARE*), *Health Technology Assessment (HTA) database*, *TRIP Database*, *PDQ-Evidence*, *Epistemonikos*, and *Health Systems Evidence*. Reviews published in English, French, or Spanish between 1 January 1995 and the search date will be considered. Structured search strategies will be developed using the thesaurus terms of each database (e.g., EMTREE for EMBASE, MeSH for MEDLINE, and PUBMED) and using free text, targeting the “title” and “abstract” fields. The strategy will then be adapted to the other databases. A health information specialist will be consulted for the development of the search strategies, and this person will help for performing the searches.

An example of a search strategy in PubMed is presented in Table 1. This strategy will be adapted and refined according to the specificities of the other databases.

**Table 1:** PubMed search strategy

PubMed	PubMed query
#1	Decision Making, Computer-Assisted/nursing [Mesh:noexp]
#2	Remote Consultation [Mesh]
#3	Telemedicine [Mesh:noexp]
#4	Telenursing [Mesh])
#5	Electronic Health Records [Mesh]
#6	Medical Records Systems, Computerized [Mesh:noexp]
#7	Public Health Informatics [Mesh]
#8	(Decision Making, Computer-Assisted/nurs* OR Remote Consultation* OR Telemedicine OR Telenursing OR Electronic Health Record* OR Medical Record* System* OR Computerized OR Public Health Informatics [Title/Abstract] OR (telehealth OR “information technolog*” OR information and communication technolog* OR personal digital assistant* [Title/Abstract])
# 9	Advanced Practice Nursing [Mesh]
# 10	Evidence-Based Nursing [Mesh]
# 11	Nursing [Subheading])
# 12	Nursing Care [Mesh:noexp])
# 13	Nursing Diagnosis [Mesh]
#14	“Nurs*” OR “Nurs* practice” OR “nursing practice”
#15	#9 OR #10 OR #11 OR #12 OR #13 OR #14
#16	Review OR systematic review [Mesh]
#17	#8 AND #15 AND #16

We will also conduct hand searches in the following journals in health informatics: *Computer Informatics Nursing*, *Journal of Telemedicine and Telecare*, *International Journal of Medical Informatics*, *Journal of Medical Internet Research*, *Journal of American Medical Informatics Associations*, *and Telemedicine journal*, *and E-health*.

### Data collection and analysis

#### Selection of reviews

We will first remove duplicate publications. Two reviewers will independently screen the title and abstract of the papers in order to assess their eligibility. We will obtain full text copies of publications that meet the pre-established inclusion criteria. The reviewers will compare their results and discuss them in case of discrepancies. If a consensus is not possible, arbitration with a third review author will be required.

As suggested by Smith et al. [33] and Worswick et al. [23], we will provide details of the scope of the reviews, i.e., review level information, as part of the included systematic reviews. The following information will be retrieved for each systematic review: publication year (with authors), objectives, search strategy (and date), number of included studies, number of participants, and method of analysis employed.

#### Data extraction and management

Two independent reviewers will summarize all included reviews based on suggestions in the *Cochrane Handbook of Systematic Reviews of Intervention*s [34], in the “characteristics of included reviews” table. We will extract the following information: characteristics of the population and setting (nurses, qualification, healthcare setting), type of interventions (e.g., type of ICT and service provided, context of use, frequency of use, dosage and timing of the intervention, components included in the intervention, the way the ICTs are delivered, and the population and health condition served by the ICTs), comparisons, description of outcomes (details about the specific aspects of nursing care, the way outcomes have been measured, different types of outcomes), and review limitations. We will also document the context and implementation aspects related to the interventions. We will present the data/results in summary tables and figures.

Any disagreements arising during the data extraction process will be resolved by discussion and consensus involving the two reviewers or will involve a third review author, if needed. If any information is missing or incomplete, we will try to contact the review authors. If any data cannot be obtained, then the review will be recorded as an “Included review” without data.

#### Quality assessment/methodological quality

Two reviewers will independently assess the methodological quality of each review that meets the eligibility criteria, using the Assessment of Multiple Systematic Reviews (AMSTAR) tool [35, 36]. AMSTAR is an 11-item checklist on which reviewers put a score of 1 point when the criterion is met. Items of the AMSTAR provide an assessment of methodological criteria such as the comprehensiveness of the search strategy used and whether the quality of included studies was evaluated and accounted for [37]. We will include reviews that have an AMSTAR score of 3 or above [37]. We will also report reasons and appropriate details concerning the excluded reviews.

#### Data synthesis

If possible, we will perform a meta-analysis. If we cannot, we will undertake a statistical analysis based on types of variables, as recommended by Grimshaw et al. [38], i.e., dichotomous or continuous nursing practice outcomes and dichotomous or continuous patient outcomes. Regarding the dichotomous outcomes, we will report risk ratios and their corresponding 95 % confidence intervals and *p* values, and for continuous outcomes, mean differences will be reported [39].

We will use the PRISMA (Preferred Reporting Items for Systematic Reviews and Meta-Analyses) flow diagram [40] to show the overall process of studies selection.

#### Sensitivity analysis

We will summarize the quality of the evidence regarding the most important outcomes by using the Grades of Recommendations, Assessment, Development and Evaluation (GRADE) approach [41, 42].

#### Subgroup analysis

We will categorize the reviews into subgroups, according to the type of intervention and its purpose (ICTs, such as electronic health records used for assessment, web-based intervention for health promotion purposes, etc.), setting (e.g., primary care, community based), and if possible, the effects (positive, negative, no effect) on a specific dimension of nursing care (e.g., nurses’ practice environment, nursing processes, professional satisfaction).

## Discussion

### Expected significance of this overview

Results of this overview could be used to develop a broad picture of the dimensions of nursing care that have the potential to be supported, enhanced, or constrained by the use of ICTs to provide different kinds of care and service among patients. This overview is an interesting starting point from which to compare and contrast findings of separate reviews [33] regarding the positive and negative effects of ICTs on nursing care. The conceptualization of nursing care by using a macroscopic model is useful to explain different components/indicators of the nursing care system (nursing care performance). In so doing, we can target. In so doing, we can target “different types of outcomes” that can be influenced by nursing care, that can be influenced by nursing care and that have the potential to be affected by ICT applications.

As pointed out by Becker and Oxman [34], an overview aims to summarize evidence from more than one systematic review, where different combinations could be achieved, such as variety in terms of types of interventions (ICTs), types of outcomes, and types of population. We believe that this overview can have implications for health policy, nursing care, and research. We think that if we better understand the effects of these ICTs, we could deploy strategies to facilitate their implementation and their integration in nursing care. Consequently, we could overcome their negative effects and optimize the positive ones, in order to use them to their full potential, as a tool to support nursing care and, ultimately, improve patient outcomes.

## Abbreviations

AMSTAR: assessment of multiple systematic reviews
CINHAL: Cumulative Index to Nursing and Allied Health Literature
DARE: Database of Abstracts and Reviews
EHR: electronic health records
GRADE: Grading of Recommendations, Assessment, Development, and Evaluation
HTA: health technology assessment
ICT: information and communication technology
MeSH: Medical Subject Heading
SMS: Short Message Service
PICO: participants, interventions, comparisons and outcomes
PRISMA: Preferred Reporting Items for Systematic Reviews and Meta-Analysis
